# The University of Michigan Dioxin Exposure Study: Population Survey Results and Serum Concentrations for Polychlorinated Dioxins, Furans, and Biphenyls

**DOI:** 10.1289/ehp.11780

**Published:** 2008-12-22

**Authors:** Elizabeth Hedgeman, Qixuan Chen, Biling Hong, Chiung-Wen Chang, Kristen Olson, Kathleen LaDronka, Barbara Ward, Peter Adriaens, Avery Demond, Brenda W. Gillespie, James Lepkowski, Alfred Franzblau, David H. Garabrant

**Affiliations:** 1 Department of Environmental Health Sciences and Risk Science Center and; 2 Department of Biostatistics, University of Michigan School of Public Health, Ann Arbor, Michigan, USA;; 3 Department of Sociology, University of Nebraska, Lincoln, Nebraska, USA;; 4 Survey Research Center, Institute for Social Research, University of Michigan, Ann Arbor, Michigan, USA;; 5 Department of Civil and Environmental Engineering, University of Michigan College of Engineering, Ann Arbor, Michigan, USA

**Keywords:** biomonitoring, dioxins, dust, environmental exposure, furans, polychlorinated biphenyls, serum, soil, survey

## Abstract

**Background:**

The University of Michigan Dioxin Exposure Study was undertaken to address concerns that the discharge of polychlorinated dibenzo-*p*-dioxins (PCDDs) and polychlorinated dibenzo furans (PCDFs) from the Dow Chemical Company in the Midland, Michigan, area had resulted in contamination of soils in the Tittabawassee River floodplain and the city of Midland, leading to an increase in residents’ body burdens of these compounds.

**Objective:**

In this article we present descriptive statistics from the resident survey and sampling of human serum, household dust, and soil and compare them with other published values.

**Methods:**

From a multistage random sample of populations in four areas of Midland and Saginaw counties and from a distant referent population, we interviewed 946 adults, who also donated blood for analysis of PCDDs, PCDFs, and polychlorinated biphenyls (PCBs). Samples of household dust and house perimeter soil were collected from consenting subjects who owned their property.

**Results:**

All five study populations were comparable in age, race, sex, and length of residence in their current home. Regional differences existed in employment history, personal contact with contaminated soils, and consumption of fish and game from contaminated areas. Median soil concentrations were significantly increased around homes in the Tittabawassee River floodplain (11.4 ppt) and within the city of Midland (58.2 ppt) compared with the referent population (3.6 ppt). Median serum toxic equivalencies were significantly increased in people who lived in the floodplain (23.2 ppt) compared with the referent population (18.5 ppt).

**Conclusions:**

Differences in serum dioxin concentrations among the populations were small but statistically significant. Regression modeling is needed to identify whether the serum concentrations of PCDDs, PCDFs, and PCBs are associated with contaminated soils, household dust, and other factors.

The University of Michigan Dioxin Exposure Study (UMDES) was undertaken in response to concerns from residents that the discharge of dioxin-like compounds (DLCs) from the Dow Chemical Company facilities in Midland, Michigan, resulted in contamination of soils in the Tittabawassee River floodplain and in the city of Midland, leading to an increase in residents’ body burdens of polychlorinated dibenzo-*p*-dioxins (PCDDs) and polychlorinated dibenzofurans (PCDFs).

PCDDs, PCDFs, and polychlorinated biphenyls (PCBs) are ubiquitous in soils, sediments, air, and animal tissues in industrialized nations [Alcock and Jones 1996; U.S. Environmental Protection Agency (EPA) 2003]. They are often the unintentional byproducts of burning and waste incineration, primary and secondary metal smelting, and chlorine-based chemical processes (U.S. EPA 2006). Within the lower peninsula of Michigan, background soil concentrations of the 17 PCDDs and PCDFs recognized by the World Health Organization (WHO) as having dioxin-like activity (Van den Berg et al. 1998) range from 0.4 to 34.7 ppt toxic equivalency (TEQ_DF-1998_, where D is PCDDs, F is PCDFs, and 1998 indicates the WHO 1998 toxic equivalency factors, respectively), with a median level of 4.6 ppt TEQ_DF-1998_ [Michigan Department of Environmental Quality (MDEQ) 1999]. In contrast, soils and sediments sampled from along the Tittabawassee River, downstream of the Dow Chemical Company, ranged from 4 to 1,980 ppt TEQ_DF-1998_ (Hilscherova et al. 2003). Previous sampling of the top 1 in. of soils from along the Tittabawassee River (Demond et al. 2008) ranged from 1.1 to 9,351 ppt TEQ_DFP-1998_ (or from 1.1 to 7,258 ppt TEQ_DFP-2005_; where D is PCDDs, F is PCDFs, P is PCBs, and 1998 or 2005 indicates the WHO 1998 or 2005 toxic equivalency factors, respectively). These elevated soil concentrations are the result of a century of chlorine and chlorine-based chemical manufacture.

With the increased awareness of the elevated concentrations of PCDDs and PCDFs in the Tittabawassee River floodplain soils, there has been increasing pressure to monitor the concentrations of PCDDs and PCDFs in the local biota. Fish caught from the rivers downstream of the Dow Chemical Company have concentrations of PCDDs and PCDFs ranging from 1.5 to 40 ppt wet weight TEQ_DF-2005_ (MDEQ 2003). In the *Family Fish Consumption Guide*, the Michigan Department of Community Health (MDCH) cautioned fishers to limit or avoid consumption of fish from these waters (MDCH 2004a). White-tailed deer, wild turkeys, and squirrels harvested from areas downstream of the Dow Chemical Company were reported to have a statistically elevated mean TEQ_DF-1998_ in their tissues compared with animals harvested from a reference area (Entrix, Inc. 2004). With respect to humans, a pilot study of 20 residents living on contaminated Tittabawassee River floodplain soils showed elevated mean serum levels of TEQDFP-1998 and 2,3,7,8-tetrachlorodibenzo-*p*-dioxin (TCDD), although the range of the serum samples fell within the age-specific range of the comparison population (MDCH 2007). Although various soils, fish, and animals from the Tittabawassee River floodplain and surrounding areas have increased levels of DLCs, it is unclear whether these contribute to human serum levels and by what pathways.

The UMDES is the first population-based exposure study of residents living in and around the Tittabawassee River floodplain. It was designed to determine whether residents living on contaminated soils, participating in activities in the contaminated region, and eating fish, game, and other foods from the contaminated region have higher serum levels of dioxins than residents in areas with no unusual source of DLCs. This study included a multi stage, random sample of the population who were interviewed in person, gave an 80-mL blood sample, and allowed collection of household dust and residential soil samples. We analyzed the blood serum, household dust, and soil samples for the 29 PCDD, PCDF, and PCB congeners recognized by WHO as having dioxin-like activity (Van den Berg et al. 1998, 2006). In this article, we describe the characteristics of the populations interviewed and the serum, household dust, and house perimeter soil concentrations determined by high-resolution gas chromatography/high-resolution mass spectrometry analyses for PCDDs, PCDFs, and PCBs. With detailed serum concentration data from 946 individuals, the UMDES is one of the largest population studies of human exposure to PCDDs, PCDFs, and PCBs in the United States.

## Methods

The methods are described more in detail by Garabrant et al. (2009b). The study was approved by the University of Michigan internal review board, and participant confidentiality is further protected by a Certificate of Confidentiality from the National Institutes of Health (Bethesda, MD).

### Study population

Residents of Midland and Saginaw counties and southwestern Bay County, Michigan, were eligible for selection to participate. Residents of Jackson and Calhoun counties (~ 100 mi south of Midland) were chosen as a referent population because those counties had no known unusual source of DLCs. Data obtained from the 2000 U.S. Census before data collection indicated that the combined population of Jackson and Calhoun counties was comparable to the population of Midland and Saginaw counties in terms of age distribution, sex, racial makeup, proportion employed in manufacturing industries, and mixture of urban and rural communities (U.S. Census Bureau 2000). We categorized the populations of these five counties into five geographically defined regions, based on the locations of their residences: *a*) residents of Midland and Saginaw counties who resided in the floodplain of the Tittabawassee River downstream of the Dow facility (referred to as “floodplain” subjects); *b*) residents of Midland and Saginaw counties who resided in areas adjacent to the floodplain of the Tittabawassee River downstream of the Dow facility (near floodplain); *c*) residents of Midland, Saginaw, and Bay counties who did not reside in or near the floodplain of the Tittabawassee River or any other river (other Midland/Saginaw); *d*) residents of Midland who lived downwind of the Dow facility in the area of aerosol emissions deposition as defined by environmental modeling (Midland plume) (Goovaerts et al. 2007); and *e*) residents of Jackson and Calhoun counties (Jackson/Calhoun).

Adults ≥ 18 years of age who had lived in their current residence for ≥ 5 years were eligible to participate. Data were collected in 2004–2005. All participants gave written informed consent before participation. A total of 946 participants completed the interview and gave a blood sample; 746 donated household dust, and 766 donated house perimeter soil.

### Interviews

All participants were interviewed in person using a standardized questionnaire that included information on demographics; pregnancy and breast-feeding; smoking; residential history; property use; work history; recreational activities; past consumption of meat, fish, poultry, and dairy; and current diet. Participants were asked to recall activities and events over their entire lifetimes. Responses could be summed over years (duration) and/or dichotomized (ever/never). For example, participants who answered yes to the question “Have you ever worked at a foundry?” would be prompted to indicate which years over their lifetime they had been employed at a foundry; this answer could then be dichotomized as ever/never worked at a foundry over their lifetime, or analyzed as total number of years worked at a foundry over their lifetime. Most activity data presented here is dichotomized as lifetime ever/never.

### Collection and laboratory analyses

Blood was collected using standard medical procedures; household dust collection methods are described in detail by Garabrant et al. (2009b); and house perimeter soil collection methods have been described by Demond et al. (2008). We shipped blood serum, household dust, and soil samples to Vista Analytical Laboratories (El Dorado Hills, CA) for analysis. Vista performed analyses for the 29 PCDDs, PCDFs, and PCBs for which consensus toxic equivalency factors (TEFs) have been published (Van den Berg 2006), using modified U.S. EPA protocols 8290 (U.S. EPA 1994) and 1668 (U.S. EPA 1999). For serum samples, total lipids were calculated from measurements of triglycerides and total cholesterol (Phillips et al. 1989). All serum dioxin concentrations are given in picograms per gram lipid (equivalent to parts per trillion), and household dust and soil data are presented as picograms per gram dry weight (equivalent to parts per trillion).

### Statistical analyses

All descriptive statistics (means, SEs, medians, frequencies) were calculated by incorporating the complex survey sample design (clustering, stratification, and unequal weighting). Because this study was based on population sampling and because all analyses incorporate the survey sample design, inferences from the analyses apply to the populations in the five regions. All statistical analyses were performed using SAS version 9.12 (SAS Institute Inc., Cary, NC). We calculated differences between population means using *t*-tests (proc surveyreg function in SAS) and analyzed differences between population frequencies using Wald chi-square (proc surveylogistic). For TEQ, we used chi-square to test whether the proportion of samples in each Midland/Saginaw region above the median of the Jackson/Calhoun referent population was different from 0.5. Toxic equivalencies (TEQs) were calculated using the 2005 WHO TEFs (TEQ_2005_) (Van den Berg et al. 2006) or the 1998 WHO TEFs (TEQ_1998_) (Van den Berg et al. 1998), as specified. We estimated individual congener concentrations falling below the limit of detection (LOD) using 
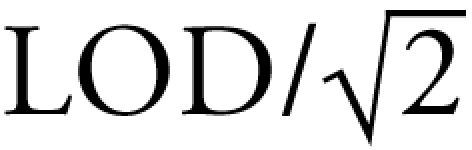
 (Hornung and Reed 1990); for comparison purposes, we also calculated one set of serum TEQs with concentrations < LOD assigned as 0. The medians of individual congeners were also calculated using the reverse Kaplan-Meier (or left-censored Turnbull) estimator (Turnbull 1976) [see Supplemental Material (available online at http://www.ehponline.org/members/2008/11780/suppl.pdf

## Results

Demographic factors, duration of residence, and history of pregnancy, breast-feeding, and smoking for the participants of each of the five populations are presented in Table 1. The four Midland/Saginaw populations were similar in age to the Jackson/Calhoun population, with the exception of the floodplain, where individuals were slightly older (53.8 years in floodplain vs. 49.9 years in Jackson/Calhoun, *p* < 0.05). We found no significant differences in body mass index (BMI) among the populations, although the floodplain population reported a smaller BMI loss in the past 12 months than did the Jackson/Calhoun population (0.7 vs. 1.1 kg/m^2^, *p* < 0.05). The near-floodplain population had smoked less, and women from the near-floodplain population had breast-fed less, than women in the Jackson/Calhoun population (*p* < 0.05). All populations were predominantly white (85–99.7%). The proportion of females in the floodplain (54.6%) and in the Midland plume (72.4%) populations were not significantly different than in the Jackson/Calhoun population (61.9%), whereas the proportion of females was significantly lower in the near-floodplain (46.0%) and other Midland/Saginaw (51.3%) populations than in the Jackson/Calhoun population. The five populations had lived in their current homes for an average of 15–20 years, had lived in their respective counties for an average of 35–44 years, and had rarely moved between Midland/Saginaw and Jackson/Calhoun counties. Thus, the populations had substantial duration of exposure to various factors in their home regions and little exposure outside of these regions.

Substantial proportions of the populations in the floodplain and Midland plume regions reported their homes had been flooded by the Tittabawassee River (38.2% and 15.2%, respectively; Table 2). Fewer than 3% of the remaining Midland/Saginaw populations experienced residential flooding by the Tittabawassee River. The floodplain and near-floodplain populations reported using weed killers for significantly more years over their lifetimes and burning trash for significantly fewer years than did the population of Jackson/Calhoun counties. On average, the other Midland/Saginaw population spent significantly more time living on a farm and using a fireplace or wood-burning stove than did the Jackson/Calhoun population. The near-floodplain population was significantly less likely to have worked in a flower garden (36.6%) than the Jackson/Calhoun population (49.5%).

The work history section of the questionnaire focused on occupations with potential exposure to PCDDs, PCDFs, and/or PCBs (Table 3). In Midland and Saginaw counties, 8.0–11.1% of the population reported having been employed at the Midland Dow facility. The proportion of the population that had lived with someone who was employed at Dow ranged from 63.8% in the Midland plume to 8.6% in the near-floodplain, to 0% in Jackson/Calhoun. The other Midland/Saginaw, near-floodplain, and floodplain region populations were as likely as the Jackson/Calhoun population to report having ever worked at a foundry. Five percent or less of the population in any region reported having ever sprayed herbicides professionally; worked in waste disposal, water treatment, pulp or paper mills, or scrap yards; or ever been stationed in Vietnam for the military.

We found no significant differences in population participation in hunting, fishing, and water activities (e.g., swimming, boating, hiking, picnicking) in Michigan outside of the five-county study area (Table 4). However, the Midland/Saginaw populations were significantly more likely than the Jackson/Calhoun population to hunt, fish, and/or participate in water activities around the Tittabawassee River, Saginaw River, and Saginaw Bay. More than one-third of each of the four Midland/Saginaw populations participated in fishing or water activities around these water bodies, indicating that a substantial proportion of the population recreated in the contaminated areas.

Corresponding with the above recreational activities, the Midland/Saginaw populations were significantly more likely than the Jackson/Calhoun population to have ever consumed sport-caught fish from the Tittabawassee River, Saginaw River, and/or Saginaw Bay in their lifetimes (Table 5). The Midland/Saginaw populations were also significantly more likely than the Jackson/Calhoun population to have consumed sport-caught fish and game from the Tittabawassee River, Saginaw River, and Saginaw Bay area in the past 5 years. Substantial proportions of all five populations (59.2–72.7%) had recently consumed sport-caught fish or game from outside the study regions.

Table 6 displays the TEQ_DFP-2005_ for the lipid-adjusted serum, household dust, and the top 1 in. of house perimeter soil for the five populations. The median serum TEQ_DFP-2005_ varied by 6.4 ppt among the five populations, was significantly elevated in the floodplain compared with the referent population (23.2 ppt vs. 18.5 ppt, *p* < 0.01), and was lowest in the Midland plume population (16.8 ppt). Maximum TEQ_DFP-2005_ concentrations varied by more than 130 ppt across the five populations, with the most elevated serum concentrations found in the floodplain (211 ppt) and near-floodplain (154 ppt) populations, and the lowest maximum serum concentration in the Midland plume (78.5 ppt) population. Median household dust concentrations were significantly elevated in the homes of the Midland plume population compared with homes of the Jackson/Calhoun population (31.3 ppt vs. 13.8 ppt, *p* < 0.01). Median house perimeter soil TEQ_DFP-2005_ levels were significantly elevated around homes in the floodplain (11.4 ppt, *p* < 0.01), Midland plume (58.2 ppt, *p* < 0.01), and other Midland/Saginaw (5.3 ppt, *p* < 0.05) populations compared with homes of the Jackson/Calhoun population (3.6 ppt). We found elevated maximum soil concentrations around the homes of the floodplain, near-floodplain, and Midland plume populations (1,881, 2,300, and 746 ppt TEQ_DFP-2005_, respectively). A complete review and discussion of the soil sample results have been reported previously by Demond et al. (2008).

The medians and ranges of lipid-adjusted serum concentrations for the 29 dioxin-like congeners are presented in Table 7. Corresponding with the serum TEQs, individual congener concentrations were right skewed. Only a small proportion of the blood samples were < LOD. However, 6 PCDF congeners had substantial proportions of samples < LOD: 2,3,7,8-tetrachlorodibenzofuran (TCDF; 68.2%), 1,2,3,7,8-penta chlorinated dibenzofuran (PeCDF; 75.7%), 1,2,3,7,8,9-hexachlorinated dibenzofuran (HxCDF; 99.6%), 2,3,4,6,7,8-HxCDF (56.9%), 1,2,3,4,7,8,9-hepta-chlorinated dibenzofuran (HpCDF; 99.3%), and octachlorinated dibenzofuran (OCDF; 89.6%). For PCB-81, 46.6% of the samples were < LOD. Of the 5 congeners that contribute 76.5% to the serum TEQ_DFP-2005_ (i.e., 2,3,7,8-TCDD, 1,2,3,7,8-PeCDD, 1,2,3,6,7,8-HxCDD, 2,3,4,7,8-PeCDF; and PCB-126), only for TCDD were > 2% of observations < LOD (13.2%). The serum congener detection results correspond with serum data from other population studies (Bates et al. 2004; Ferriby et al. 2007; Wong et al. 2008). Thus, the present study had good ability to estimate the population distribution for almost all of the DLC congener concentrations.

In Table 8 we present percentile distributions of the serum TEQ_DFP-1998_ and TEQ_DFP-2005_ compared with the National Health and Nutrition Examination Survey (NHANES) data representing the concentrations of DLCs in the U.S. adult population (Ferriby et al. 2007; Patterson et al. 2008). The population serum data from the present study bracket the NHANES 2001–2002 serum data published by Ferriby et al. (2007). With the exception of the maximum TEQ_DFP-1998_ observation, serum percentiles from the Jackson/Calhoun population vary from the U.S. adult population by < 5 ppt. Within the Midland and Saginaw county populations, serum concentrations are similar to the NHANES 2001–2002 data up to the 50th percentile, but then diverge from the U.S. population data with an increase of 10–30 ppt.

## Discussion

A central goal of the UMDES was to determine whether populations living in an area with elevated concentrations of DLCs experienced a higher level of exposure than do populations living in areas with background concentrations of these compounds. We selected residents of Jackson and Calhoun counties as the comparison population because their demographics were similar to those of residents of Midland and Saginaw counties and because they had no known unusual source of dioxin exposure.

Corresponding with the data obtained from the U.S. Census, our data indicate that, with few exceptions, the populations were similar in age, race, and sex. We found few significant differences among the populations with respect to smoking habits, live births and breast-feeding history, or length of residence in their current home. Moreover, our data indicate that the populations were stable and had lived in their respective regions for most of their lifetimes. With the exception of individuals living in the Tittabawassee River floodplain region, the populations of Midland and Saginaw counties and Jackson and Calhoun counties were equally likely to have hunted and consumed game, or fished and consumed fish from areas outside of their specific home region. Also, with few exceptions, they were equally likely to have worked in a number of occupations not associated with the Dow Chemical Company.

The primary difference between the Midland/Saginaw populations and Jackson/Calhoun populations was the presence of the Dow Chemical Company and the elevated soil concentrations of PCDDs and PCDFs in the surrounding area. Our results showed significant regional differences in employment with the Dow Chemical Company, personal contact with contaminated soils and sediments, and consumption of fish and game from contaminated areas. Less than 1% of individuals living in Jackson and Calhoun counties had ever been employed at the Midland Dow Chemical facility, whereas 8.0–11.1% of individuals from Midland and Saginaw had worked at the facility. Despite warnings published by the MDCH (2004a, 2004b), 2.6–8.5% of the Midland and Saginaw populations had consumed fish from the Tittabawassee River in the past 5 years, 17.9–28.7% had consumed fish from the Saginaw River or Bay in the past 5 years, and 9.5–18.3% had consumed game from the Tittabawassee River floodplain in the past 5 years.

The UMDES questionnaire included a set of questions concerning activities on the Kalamazoo River between the Morrow Pond Dam and Lake Michigan because this area is known to have elevated concentrations of PCBs from paper mills (U.S. EPA 2007). This segment of the Kalamazoo River begins about 10 miles downstream of the western boundary of Calhoun County, outside of our study communities. Our data indicate that the Jackson/Calhoun population and a small number of participants in the Midland and Saginaw populations participate in activities around this area of the Kalamazoo River. However, ≤ 1% of adults from any of the five populations had consumed fish from this area in the past 5 years.

The median serum TEQ_DFP-2005_ for residents of the floodplain population was 5 ppt higher than for the Jackson/Calhoun referent population; this elevation was statistically significant, indicating an upward shift in the population body burden. This elevation is also apparent in the percentile distribution data presented in Table 8. Of the five congeners that contributed most to the serum TEQ, three are of particular interest because of their elevation in soil samples from areas believed to be contaminated by Dow. The primary contributor to the TEQ from soil around homes in the Tittabawassee River floodplain was 2,3,4,7,8-PeCDF (Demond et al. 2008; Hilscherova et al. 2003). Soil samples taken from around homes located in the aerosol deposition plume downwind of the Dow facility were primarily elevated in 2,3,7,8-TCDD and 1,2,3,7,8-PeCDD (Demond et al. 2008). The data presented in Table 6 show that the median serum values of 2,3,7,8-TCDD, 1,2,3,7,8-PeCDD, and 2,3,4,7,8-PeCDF vary by no more than 2.2 ppt each across the five populations. However an elevation in maximum serum concentration of TCDD is apparent when comparing the Midland and Saginaw county populations with the Jackson and Calhoun county population; this result is consistent across all of the Midland/Saginaw regions (Table 6). There is also an apparent elevation in the maximum serum concentrations of 1,2,3,7,8-PeCDD and 2,3,4,7,8-PeCDF, although these results are less consistent across the Midland and Saginaw populations. A discussion of subjects with elevated serum concentrations of these congeners has been previously published (Franzblau et al. 2008a, 2008b).

Overall, our data do not vary substantially from other recent national publications of serum DLC concentrations in adults. In their comprehensive analyses of NHANES 2001–2002 data, Ferriby et al. (2007) and Patterson et al. (2008) identified only a 3–4 ppt difference between both the median and 95th percentile serum TEQ_DFP-1998_ in the U.S. population and the UMDES referent population data presented here. New 95th percentile serum TEQ_DFP-1998_ from NHANES 2003–2004 are 15–30 ppt lower than both the NHANES 2001–2002 data and the UMDES data, although the reason for this 20–25% decrease in TEQ_DFP-1998_ is unclear (Patterson et al. 2008). In a 2002 study of residential exposure to local industries in Louisiana, Wong et al. (2008) reported differences in serum geometric means for their exposed and referent populations of < 1 ppt TEQ_DFP-1998_; their data differed only 5–10 ppt TEQ_DFP-1998_ from the UMDES data. These population serum concentrations contrast with the median (447 ppt TCDD) and maximum (56,000 ppt TCDD) observed serum levels of Seveso, Italy, residents who lived near the Industrie Chimiche Meda Società Azionaria (ICMESA) 2,4,5-trichlorophenol plant when it exploded in 1976 (Needham et al. 1997/1998).

Although exposure of the general population to PCDDs, PCDFs, and PCBs is modeled to occur predominantly via ingestion of foodstuffs (Schecter et al. 2001; U.S. EPA 2003), there has been little investigation into the actual proportion of human exposure that occurs through proximity to contaminated soils. The UMDES is a large, population-based study designed to determine whether residents living on contaminated soils, participating in activities in the contaminated region, and eating fish, game, and other foods from the contaminated region have higher serum levels of dioxins than do residents in areas with no unusual source of DLCs. Our study has shown small but statistically significant elevations in median serum TEQ_DFP-2005_ for residents living in areas with elevated soil concentrations of PCDDs and PCDFs. The specific route(s) of exposure is not identifiable from this assessment of body burden but will be discussed in future publications.

## Figures and Tables

**Table 1:** Demographic characteristics (mean ± SE or percent) of residents in the five study populations.

Characteristic	Midland and Saginaw counties				Jackson and Calhoun counties (*n* = 251)
	Floodplain (*n* = 251)	Near floodplain (*n* = 197)	Midland plume (*n* = 48)	Other (*n* = 199)	
Age (years)	53.8 ± 1.1*	50.9 ± 1.3	50.3 ± 2.0	52.7 ± 2.0	49.9 ± 1.3
BMI (kg/m^2^)	27.8 ± 0.4	27.5 ± 0.5	29.5 ± 1.2	29.1 ± 0.7	28.7 ± 0.5
BMI loss (kg/m^2^) in past 12 months	0.7 ± 0.1*	0.9 ± 0.3	0.8 ± 0.3	1.1 ± 0.3	1.1 ± 0.1
Cigarette pack-years	12.7 ± 1.4	8.0 ± 1.3*	10.7 ± 3.2	12.4 ± 1.6	12.5 ± 1.4
No. of live births^a^	1.9 ± 0.2	2.0 ± 0.2	2.6 ± 0.3	2.1 ± 0.2	2.3 ± 0.2
Duration of breast-feeding (months)^a^	5.3 ± 1.4	3.8 ± 1.0*	9.5 ± 2.8	5.6 ± 2.3	6.5 ± 1.0
Years at current residence	18.9 ± 0.8*	15.4 ± 0.8	19.2 ± 2.7	20.4 ± 1.5*	15.9 ± 1.0
Total years in Midland/Saginaw	44.0 ± 1.5**	37.6 ± 2.0**	35.0 ± 2.6**	39.7 ± 2.1**	0.1 ± 0.1
Total years in Jackson/Calhoun	0.3 ± 0.2**	0.8 ± 0.7**	0 ± 0**	0.2 ± 0.2**	38.0 ± 1.2
Frequency (%)					
White	99.7**	94.1	98.8	89.5*	95.2
Female	54.6	46.0**	72.4	51.3*	61.9
High school graduate	91.7	94.3*	92.3	87.0	86.2

a Among women only.

* *p* < 0.05, and

** *p* < 0.01, compared with Jackson/Calhoun.

**Table 2:** Property use (mean ± SE or percent) by residents from the five study populations.

Use category	Midland and Saginaw counties				Jackson and Calhoun counties (*n* = 251)
	Floodplain (*n* = 251)	Near floodplain (*n* = 197)	Midland plume (*n* = 48)	Other (*n* = 199)	
Total years using weed killers around the home before 1960	10.9 ± 1.2**	10.2 ± 1.2**	7.8 ± 2.4	7.7 ± 0.8	5.8 ± 0.7
Total years burning trash	8.7 ± 0.8*	7.9 ± 1.0**	7.9 ± 2.6	15.2 ± 1.2	12.4 ± 1.4
Total years residence on farm (crop, livestock, poultry)	4.1 ± 0.6*	4.5 ± 0.9	4.5 ± 1.5	9.3 ± 1.4*	6.0 ± 0.7
Total years using a fireplace and/or woodstove	9.5 ± 1.0	10.4 ± 1.0	10.2 ± 2.0	13.8 ± 1.3**	9.4 ± 0.9
Total years in a fire-damaged home	0.2 ± 0.0	0.1 ± 0.0	0.3 ± 0.2	0.2 ± 0.0	0.3 ± 0.1
Frequency (%)					
Residence ever flooded by the Tittabawassee River	38.2**	2.7**	15.2**	2.2**	0
Personally worked in a flower garden	42.2	36.6*	63.2	45.8	49.5

* *p* < 0.05, and

** *p* < 0.01, compared with Jackson/Calhoun.

**Table 3:** Work history of residents from the five study populations [frequency (%)].

Characteristic	Midland and Saginaw counties				Jackson and Calhoun counties (*n* = 251)
	Floodplain (*n* = 251)	Near floodplain (*n* = 197)	Midland plume (*n* = 48)	Other (*n* = 199)	
Ever employed at the Midland Dow chemical facility	9.7**	8.0**	9.8**	11.1**	0.5
Ever lived with someone while they were employed at the Midland Dow chemical facility	10.2**	8.6**	63.8**	14.1**	0.0
Ever employed at a foundry	11.3	13.2	0.7**	10.3	9.7
Ever employed as an emergency responder	5.0	6.0	12.2	4.1	6.0
Ever employed at a pulp or paper mill	2.0	2.2	3.6	2.2	2.4
Ever stationed in Vietnam for the military	2.0	2.4	0**	1.7	2.8

* *p* < 0.05, and

** *p* < 0.01, compared with Jackson/Calhoun.

**Table 4:** Recreational activities of residents from the five study populations [frequency (%)].

Characteristic	Midland and Saginaw counties				Jackson and Calhoun counties (*n* = 251)
	Floodplain (*n* = 251)	Near floodplain (*n* = 197)	Midland plume (*n* = 48)	Other (*n* = 199)	
Ever fished the Tittabawassee River	32.6**	23.5**	8.4*	22.3**	1.0
Ever fished the Saginaw River	20.8**	27.3**	10.0*	27.0**	2.0
Ever fished the Saginaw Bay	43.1**	46.8**	39.5**	46.0**	4.3
Ever fished the Kalamazoo River^a^	0**	0**	9.8	1.1*	7.0
Ever fished any other Michigan waters	74.0	71.4	88.7	76.3	76.1
Ever hunted the Tittabawassee River floodplain below the Tridge	12.3**	10.7**	1.4	6.6*	1.0
Ever hunted the surrounding areas of the Saginaw River	4.6*	9.9**	6.5*	4.1*	0.5
Ever hunted the surrounding areas of the Saginaw Bay	5.8**	9.6**	10.6*	3.7*	0.3
Ever hunted the surrounding areas of the Kalamazoo River^a^	0**	0**	9.6	0.9	1.3
Ever hunted any other Michigan water plains	17.3	26.4	21.9	27.4	18.4
Ever did water activities near the Tittabawassee River^b^	44.4**	30.7**	18.2**	21.6**	1.9
Ever did water activities near the Saginaw River^b^	43.3**	49.0**	15.2*	28.8**	2.2
Ever did water activities near the Saginaw Bay^b^	60.1**	59.3**	45.3**	46.7**	5.7
Ever did water activities near the Kalamazoo River^a^,^b^	0**	1.2**	9.8	1.4**	13.0
Ever did water activities near any other Michigan waters^b^	83.7	81.0	90.9	81.9	85.2

Tridge, a three-way footbridge in downtown Midland.

a Between the Morrow Pond Dam and Lake Michigan.

b Includes swimming, boating, hiking, and so forth.

* *p* < 0.05, and

** *p* < 0.01, compared with Jackson/Calhoun.

**Table 5:** Dietary habits of residents from the five study populations [frequency (%)].

Characteristic	Midland and Saginaw counties				Jackson and Calhoun counties (*n* = 251)
	Floodplain (*n* = 251)	Near floodplain (*n* = 197)	Midland plume (*n* = 48)	Other (*n* = 199)	
Ever consumed fish from the Tittabawassee River, Saginaw River, or Saginaw Bay	63.2**	55.8**	46.0**	45.9**	7.4
Ever consumed fish from the Kalamazoo River^a^	0**	0.3**	9.8	1.5*	6.7
Consumed fish from the Tittabawassee River in the past 5 years	8.5**	3.6**	2.6*	5.3**	0.2
Consumed fish from the Saginaw River or Bay in the past 5 years	17.9**	28.7**	20.3**	19.2**	2.6
Consumed fish from the Kalamazoo River in the past 5 years^a^	0**	0**	0**	0.8	1.0
Consumed fish from any other river in the past 5 years	59.2**	63.0	61.0	69.2	72.7
Consumed game from the Tittabawassee River floodplain in the past 5 years	18.3**	11.5**	14.3**	9.5**	0.4
Consumed game from the Saginaw River or Bay floodplain in the past 5 years	7.7**	9.2**	4.2**	8.7**	0
Consumed game from any other location in the past 5 years	42.9*	57.3	54.9	56.8	56.3
Ever been a vegetarian	2.8	1.9	2.9	5.2	5.0

a Between the Morrow Pond Dam and Lake Michigan.

* *p* < 0.05, and

** *p* < 0.01, compared with Jackson/Calhoun.

**Table 6:** Median (range) TEQ_DFP-2005_ (ppt) for UMDES lipid-adjusted serum, household dust, and house perimeter soil samples.

Sample	No.	Midland and Saginaw counties				Jackson and Calhoun counties
		Floodplain	Near floodplain	Midland plume	Other	
Serum	946	23.2** (4.7–211)	21.9 (4.2–154)	16.8 (3.8–78.5)	20.7 (4.1–107)	18.5 (4.7–109)
Household dust	764	16.4 (2.3–1,748)	11.3 (1.4–189)	31.3** (8.2–334)	17.6 (1.6–1,401)	13.8 (2.1–1,114)
House perimeter soil, top 1 in.	766	11.4** (1.1–1,881)	3.9 (0.8–2,300)	58.2** (6.3–746)	5.3* (0.8–158)	3.6 (0.4–186)

* *p* < 0.05, and

** *p* < 0.01, compared with Jackson/Calhoun.

**Table 7:** Median (range) of lipid-adjusted serum dioxin concentrations (in ppt) by region.

Compound	Percent samples < LOD	Median LOD (ppt)^a^	Midland and Saginaw counties				Jackson and Calhoun counties (*n* = 251)
			Floodplain (*n* = 251)	Near floodplain (*n* = 197)	Midland plume (*n* = 48)	Other (*n* = 199)	
PCDDs							
2,3,7,8-TCDD	13.2	0.5	2.4 (0.3–65.4)	2.1 (0.2–26.7)	1.9 (0.2–27.6)	1.9 (0.2–32.1)	1.4 (0.2–10.5)
1,2,3,7,8-PeCDD	1.3	2.1	6.3 (0.3–59.8)	5.6 (0.6–35)	5.3 (0.9–23.7)	5.7 (1.3–38.5)	5 (0.5–23.7)
1,2,3,4,7,8-HxCDD	7.3	2.6	5.2 (1.1–30.8)	5 (1.6–18.5)	4.4 (0.6–15)	5.3 (0.9–23.6)	5.2 (0.5–22.8)
1,2,3,6,7,8-HxCDD	0.4	3.4	39.6 (6.5–189)	34.6 (3–168)	36.6 (6–95.7)	40.4 (2.4–174)	39.4 (1.2–163)
1,2,3,7,8,9-HxCDD	6.1	2.6	6.5 (1.3–34.3)	5.8 (0.4–20.7)	5 (0.7–16.4)	6.3 (0.8–31.7)	6.4 (0.7–41.3)
1,2,3,4,6,7,8-HpCDD	0.2	2.1	37.7 (1.6–430)	38.6 (8.3–133)	35.1 (8–146)	39 (8.5–253)	32.7 (1.5–201)
OCDD	0	NA	246 (40.6–3,270)	230 (51.2–1,200)	211 (65.6–1,580)	245 (55.1–1,720)	249 (35.3–1,850)
PCDFs							
2,3,7,8-TCDF	68.2	0.4	0.3 (0.1–5.5)	0.3 (0.1–7.4)	0.3 (0.1–3.9)	0.3 (0–4.9)	0.3 (0.1–4.2)
1,2,3,7,8-PeCDF	75.7	0.4	0.3 (0.1–25.1)	0.4 (0.1–6.1)	0.2 (0.1–5.1)	0.3 (0.1–4.9)	0.4 (0.1–4.8)
2,3,4,7,8-PeCDF	1.0	1.5	6.4 (0.3–50)	6.8 (1–33.9)	4.6 (1.3–19)	6 (0.9–29.8)	5.4 (0.8–26.2)
1,2,3,4,7,8-HxCDF	4.0	2.2	6.1 (1.4–27.7)	5.9 (1.2–19.2)	5.1 (1–12.8)	5.6 (0.6–21.6)	5.5 (0.8–23.6)
1,2,3,6,7,8-HxCDF	3.7	1.8	5.4 (0.3–96)	5.8 (0.7–36.8)	4.4 (0.6–11.1)	5.2 (1–18)	5.6 (0.7–26.1)
1,2,3,7,8,9-HxCDF	99.6	1.1	0.6 (0.1–5.6)	0.6 (0.1–6.5)	0.8 (0.1–10.4)	0.7 (0.1–7.6)	0.8 (0.2–5)
2,3,4,6,7,8-HxCDF	56.9	1.0	1 (0.1–8.5)	1 (0.2–5.8)	0.7 (0.3–6.8)	1.1 (0.1–7.5)	0.8 (0.1–6.5)
1,2,3,4,6,7,8-HpCDF	5.5	1.9	6.7 (0.9–199)	7.3 (0.8–40.7)	6.1 (1.3–10.6)	7.6 (0.7–71.5)	7.4 (0.4–89.1)
1,2,3,4,7,8,9-HpCDF	99.3	1.0	0.5 (0.1–11.3)	0.6 (0.1–5.4)	0.5 (0.2–14.8)	0.6 (0.1–7.6)	0.7 (0.2–7.4)
OCDF	89.6	2.5	1.4 (0.4–37.5)	1.7 (0.3–114)	1.2 (0.5–31.3)	1.7 (0.3–21.3)	2 (0.5–28.5)
PCBs							
PCB-77	1.6	1.0	4.1 (0.6–24.9)	3.8 (0.8–39.7)	3.3 (1.7–36.8)	4 (0.5–11.6)	4.1 (0.6–21.7)
PCB-81	46.6	1.4	1.6 (0.4–17.3)	1.6 (0.3–16.6)	1.5 (0.5–11.3)	1.3 (0.4–29.6)	1.3 (0.3–20.1)
PCB-126	1.0	4.7	22.6 (1.9–339)	24.6 (4–378)	16.3 (2.4–123)	17.9 (3.2–270)	16.5 (2.7–394)
PCB-169	0.3	3.7	25.6 (2.5–119)	28.1 (2.1–207)	19.9 (3.7–66.9)	23.7 (2.6–145)	20 (2.6–102)
PCB-105	0.1	NA	1,800 (13.7–25,600)	2,190 (124–47,000)	1,200 (258–12,300)	1,960 (252–25,400)	1,460 (0–33,300)
PCB-114	0.1	NA	1,280 (7.8–9,510)	1,300 (71.6–25,200)	1,010 (177–6,180)	1,150 (123–9,950)	1,020 (0–9,780)
PCB-118	0.1	NA	10,200 (79.6–97,100)	11,700 (892–246,000)	5,820 (1,640–60,400)	10,100 (1,500–90,600)	7,750 (0–150,000)
PCB-123	2.2	34.4	165 (1.3–2,010)	195 (14.4–3,790)	87.7 (13.6–1,080)	150 (25.8–2,610)	106 (0–2,500)
PCB-156	0.1	NA	8,460 (41.9–45,400)	8,530 (265–105,000)	7,020 (1,050–30,500)	7,410 (481–47,600)	8,630 (0–50,800)
PCB-157	0.1	NA	1,900 (8.6–9,970)	1,980 (58.3–22,800)	1,340 (225–7,400)	1,750 (120–11,000)	1,960 (0–11,700)
PCB-167	0.1	NA	1,770 (9.5–11,700)	1,860 (92.9–26,500)	1,420 (239–8,100)	1,610 (114–12,700)	1,350 (0–16,100)
PCB-189	0.1	NA	653 (3.1–2,930)	732 (16–4,920)	478 (47.8–1,970)	634 (34.7–4,110)	530 (0–3,010)

NA, not applicable. Values < LOD estimated with 
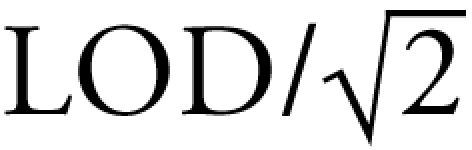
. For all congeners with < 98% of samples < LOD, the reverse Kaplan-Meier estimate of the median was either less than the 
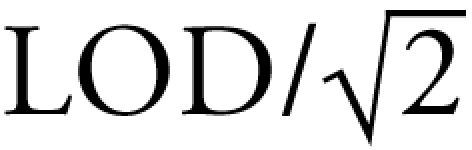
 estimate or no more than 1% greater than the 
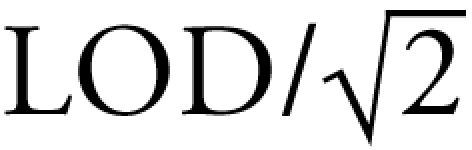
 estimate.

a Median LOD is not provided when only one or no observation was < LOD.

**Table 8:** Comparison of serum TEQs in UMDES and United States (NHANES) populations.

TEQ calculation method/nondetect assignment	Population	No.	Percentile distribution of serum TEQ (ppt)						
			Minimum	5th	25th	50th	75th	95th	Maximum
TEQ_DFP-1998_ (nondetects = 0)	Midland and Saginaw	695	3.4	9.1	17.6	27.1	44.0	82.9	238
	Jackson and Calhoun	251	2.2	7.2	15.8	23.8	35.5	66.4	150
TEQ_DFP-1998_ ( 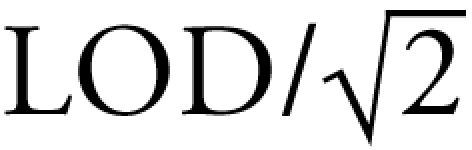 )	Midland and Saginaw	695	4.7	10.1	17.8	27.3	44.1	83.0	238
	Jackson and Calhoun	251	5.2	8.6	15.9	24.8	36.2	66.5	150
	NHANES 2001–2002^a^	1,081	8.0	NA	17.7	23.6	35.0	69.3	208.1
	NHANES 2003–2004^b^	1,237	NA	NA	NA	NA	NA	52.3	NA
TEQ_DF-1998_ ( 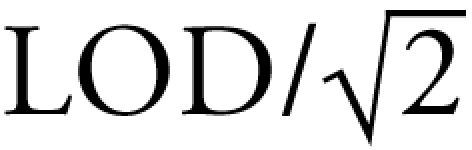 )	Midland and Saginaw	695	3.0	7.4	11.9	18.6	29.1	48.5	182.9
	Jackson and Calhoun	251	4.4	6.2	11.2	16.0	21.1	38.6	77.1
TEQ_P-1998_ ( 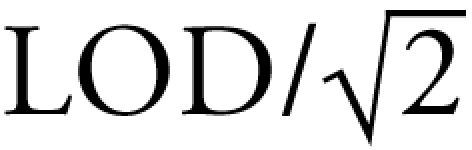 )	Midland and Saginaw	695	1.0	2.2	5.0	9.0	15.9	35.5	137.2
	Jackson and Calhoun	251	0.8	1.9	4.8	8.9	14.1	31.3	74.2
TEQ_DFP-2005_ ( 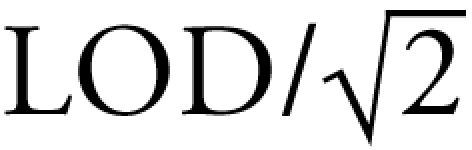 )	Midland and Saginaw	695	3.8	8.7	13.8	20.7	32.3	62.9	211
	Jackson and Calhoun	251	4.7	6.9	12.4	18.5	25.3	46.5	109
	NHANES 2003–2004^b^	1,237	NA	NA	NA	NA	NA	39.9	NA
TEQ_DF-2005_ ( 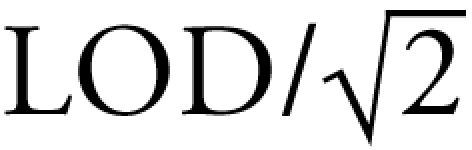 )	Midland and Saginaw	695	2.7	7.0	11.1	17.4	26.8	45.5	173
	Jackson and Calhoun	251	4.1	5.9	10.2	15.1	19.7	36.0	72.2
TEQ_P-2005_ ( 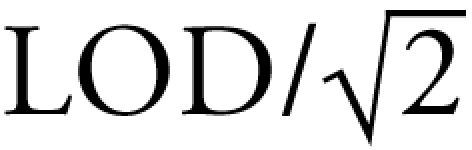 )	Midland and Saginaw	695	0.5	1.2	2.1	3.4	6.1	17.4	54
	Jackson and Calhoun	251	0.5	0.9	1.9	2.9	5.2	12.6	46.8

NA, not available.

a Data from Ferriby et al. (2007).

b Data from Patterson et al. (2008).
